# Diagnostic Accuracy and Detection Rate of Glaucoma Screening with Optic Disk Photos, Optical Coherence Tomography Images, and Telemedicine

**DOI:** 10.3390/jcm11010216

**Published:** 2021-12-31

**Authors:** Alfonso Anton, Karen Nolivos, Marta Pazos, Gianluca Fatti, Miriam Eleonora Ayala, Elena Martínez-Prats, Oscar Peral, Vladimir Poposki, Evangelos Tsiroukis, Antonio Morilla-Grasa, Merce Comas, Xavier Castells

**Affiliations:** 1 Research Department, Institut Català de la Retina (ICR), 08017 Barcelona, Spain; frutalito@hotmail.com (M.E.A.); 96535@parcdesalutmar.cat (V.P.); vagakos@hotmail.com (E.T.); amorilla@icrcat.com (A.M.-G.); 2 Medical School, Universitat Internacional de Catalunya, 08017 Barcelona, Spain; 3 Ophthalmology Service, Parc de Salut Mar, 08005 Barcelona, Spain; gianlucafatti@hotmail.com; 4 Department of Medicine, Institut Mar d’Investigacions Mèdiques (IMIM), 08005 Barcelona, Spain; karenolivos@yahoo.es; 5 Epidemiology and Evaluation Department, Parc Salut Mar, 08005 Barcelona, Spain; 93887@parcdesalutmar.cat (M.C.); XCastells@parcdesalutmar.cat (X.C.); 6 Institut Clínic d’Oftalmologia (ICOF), Hospital Clínic de Barcelona, Institut d’Investigacions Mèdiques, 08036 Barcelona, Spain; martapazoslopez@gmail.com; 7 Cap Barceloneta, 08005 Barcelona, Spain; emartinez@perevirgili.cat; 8 Cap Villa Olimpica, 08005 Barcelona, Spain; operal@capvilaolimpica.net; 9 Health Services Research on Chronic Patients Network (REDISSEC), Instituto de Salud Carlos III, 28029 Madrid, Spain

**Keywords:** glaucoma, optical coherence tomography, retinography, telemedicine, screening

## Abstract

Purpose: The aim of this study was to evaluate the diagnostic accuracy of optical coherence tomography (OCT) and retinography in the detection of glaucoma through a telemedicine program. Methods: A population-based sample of 4113 persons was randomly selected. The screening examination included a fundus photograph and OCT images. Images were evaluated on a deferred basis. All participants were then invited to a complete glaucoma examination, including gonioscopy, visual field, and dilated fundus examination. The detection rate, sensitivity, specificity, and positive and negative predictive values were calculated. Results: We screened 1006 persons. Of these, 201 (19.9%) were classified as glaucoma suspects; 20.4% were identified only by retinographs, 11.9% only by OCT images, and 46.3% by both. On ophthalmic examination at the hospital (*n* = 481), confirmed glaucoma was found in 58 (12.1%), probable glaucoma in 76 (15.8%), and ocular hypertension in 10 (2.1%), and no evidence of glaucoma was found in 337 (70.0%). The detection rate for confirmed or probable glaucoma was 9.2%. Sensitivity ranged from 69.4% to 86.2% and specificity from 82.1% to 97.4%, depending on the definition applied. Conclusions: The combination of OCT images and fundus photographs yielded a detection rate of 9.2% in a population-based screening program with moderate sensitivity, high specificity, and predictive values of 84–96%.

## 1. Introduction

Glaucoma is among the first causes of blindness, and it is estimated that it will affect 111 million people by 2040 [1]. The prevalence of glaucoma in Spain is 2.1% in individuals older than 40 years [2], which is similar to that in other Caucasian populations [3,4]. Glaucoma screening may be cost effective if performed in high-risk populations [5]. The ideal choice or combination of tests for glaucoma screening is still unknown [6]. Tonometry would miss all normal pressure glaucomas, while short functional tests have limited sensitivity and require repetition to rule out a learning effect. Optic nerve digital images have been increasingly used in the last few decades and may be acquired in a few seconds with less need for patient cooperation than functional tests. 

Recent meta-analyses have demonstrated the high classification accuracy of imaging devices for identifying glaucoma [7,8], and some have been shown to be cost effective for glaucoma screening [9,10,11]. Nevertheless, there is limited information on telemedicine screening based on the combination of two imaging technologies and no added functional test. 

Additionally, the COVID-19 pandemic has emphasized that telemedicine for detecting and following glaucoma could be particularly useful to facilitate ophthalmic care without in-person examinations, as well as the need for protecting both patients and healthcare professionals, with barriers and continuous cleaning of instruments, the usefulness of imaging devices [12,13], and the difficulties of performing functional tests while wearing a face mask [14].

The objective of the present study was therefore to assess the accuracy of glaucoma screening with optical coherence tomography (OCT) and fundus photography in an at-risk population through a telemedicine program.

## 2. Methods 

### 2.1. Telemedicine Platform

**Figure 1:**
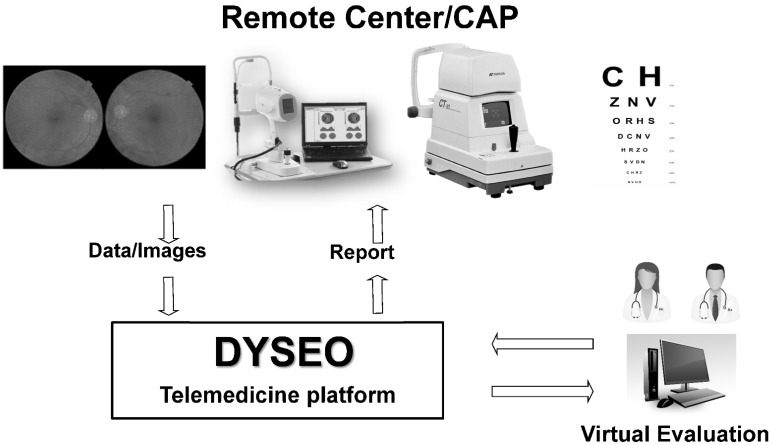
Telemedicine scheme. At the primary care center (remote center), patients were examined, and data were uploaded to the DYSEO platform. Images and data were remotely evaluated and reported on a deferred basis.

A telemedicine web-based platform designed by our team, named DYSEO and not commercially available, was customized for ophthalmic screening and implemented in an urban health area of Barcelona (Figure 1). The platform was developed with the open-source relational database management system, MySQL; was deployed in a Linux server; and complies with data protection regulations. Data were bidirectionally encrypted with secure socket layers. 

### 2.2. Sample

Screening targeted the population living in two of Barcelona’s health areas: Barceloneta and Vila Olimpica. Specifically, this population-based study targeted 18,185 males and females aged 55–85 years. A previous study conducted by our group in a similar setting encountered a low participation rate (25.5%) for several reasons [10], and, consequently, we considered a random selection of more than 4000 persons from the census necessary to obtain a sample of 1000 examinations. A detailed description of the sample calculation for the global study is described elsewhere [15]. A sample of 222 persons was estimated to be adequate, 74 screened as positive and 148 individuals screened as negative, to evaluate the diagnostic accuracy of the screening program.

### 2.3. Examinations at the Primary Care Center

All persons who agreed to participate were scheduled for an examination at the primary care center (PCC). Participants signed an informed consent form before entering the study and completed a health questionnaire. The details of the screening program are described in a previous publication [15]. Visual acuity and intraocular pressure (IOP) with an air-puff tonometer (Topcon CT80, Topcon Corporation, Oakland, NJ, USA) were also measured. The mean of two pressure measurements was used in the study. Spectrum domain OCT (SD-OCT) images of the optic disk, nerve fiber layer (NFL), and ganglion cell complex at the macula were obtained with a portable instrument (iVue, Optóvue, Freemont, CA, USA). A fundus camera (Topcon TRC, Topcon Corporation, Oakland, NJ, USA) was used to image the optic disk and the macula. Examination results were uploaded to the telemedicine platform and sent for remote evaluation. 

### 2.4. Remote Image Evaluation and Grading

A group of 16 trained ophthalmologists was asked to evaluate the quality of each image (non-evaluable, poor, fair, or good) and the presence or absence of pathological signs, globally specifying the signs identified, on easy-to-complete forms on the DYSEO platform. The specific fields to be defined by evaluators and classification methods have been previously described [15].

### 2.5. Examination at the Glaucoma Clinic of the Hospital

All participants in the screening exam performed at the PCC were invited telephonically to attend a complete ophthalmic examination at the hospital. All participants underwent anterior and posterior pole examination, visual field test, OCT images and gonioscopy. 

Visual field test was performed with Humphrey field analyzer (Carl Zeiss Meditec, Dublin, CA, USA) using the SITA Standard 24-2 test. A minimum of two reliable fields with reproducible results in the pattern deviation plot were required for global classification. Fields were repeated for up to a maximum of four exams until reproducible results were obtained. A field was considered reliable if there were less than 30% false positive responses and no artifacts. Two fields were considered reproducible if no significant improvement was present; both were normal or abnormal; or, if a significant defect was present, it was located in the same hemifield in both fields. A visual field defect was considered significant if there was a minimum of three adjacent locations outside the 95% normal limits. Finally, the visual field was considered abnormal if two adjacent fields had a significant defect and were reproducible. 

OCT images of the optic nerve head and the peripapillary retinal nerve fiber layer (RNFL) were obtained with OCT Cirrus 4000 (Carl Zeiss Meditec, Dublin, CA, USA) using the imaging protocol Optic disc cube 200 × 200. Images were only considered for classification if signal strength was 6 or above and artifacts could be ruled out after checking for image centering and delineation of retinal layers. An OCT image was considered abnormal if one or more of the following was outside 99% normal limits: average RNFL thickness, rim area, C/D ratio, or at least one RNFL 90° sector. 

Gonioscopy was evaluated with a Sussman four-mirror gonio lens and using the Shaffer classification from 0 (closed) to 4 (completely open). All examinations were performed by experienced operators.

All examinations, photographs, and test results were examined by a glaucoma specialist (AA, EA). Visual field and OCT were classified as unavailable/unreliable, normal, or abnormal in accordance with the above definitions. If the evaluator considered the test to be abnormal but not glaucomatous, the image or the field was not considered for global glaucomatous classification in this study.

The final classification was decided by the specialist in view of all the available information and with the following definitions: Persons without glaucoma had no evidence of glaucoma or ocular hypertension. Ocular hypertensives had pressure above 21 mmHg with no signs of structural or functional damage. Persons with probable glaucoma had structural or functional abnormalities compatible with glaucoma and/or occludable angle. Confirmed glaucoma patients had both functional and structural damage compatible with the disease, independently of the value of pressure or state of the angle.

### 2.6. Statistical Analysis

Data analysis was performed with free software R (version 3.4.2, Foundation for Statistical Computing, Vienna, Austria). Descriptive statistics were applied. The chi-square test was used to compare image quality between the groups, and univariate analysis was performed to assess risk factors for glaucoma in the sample. The global classification assigned by the glaucoma specialist at the hospital was used as the gold standard to calculate the detection rate, sensitivity, specificity, and positive and negative predictive values of the screening classification with remote evaluation of tests. The results of diagnostic accuracy were calculated for the three different definitions of positive diagnosis: confirmed glaucoma (definition 1); confirmed or probable glaucoma (definition 2); and, finally, confirmed or probable glaucoma or OHT (definition 3). 

### 2.7. Ethics

This study was performed in accordance with the tenets of the Declaration of Helsinki. The study protocol was approved by the ethics committee of Parc Salut Mar, and all participants signed an informed consent form before entering the study.

## 3. Results

### 3.1. Study Sample

**Figure 2:**
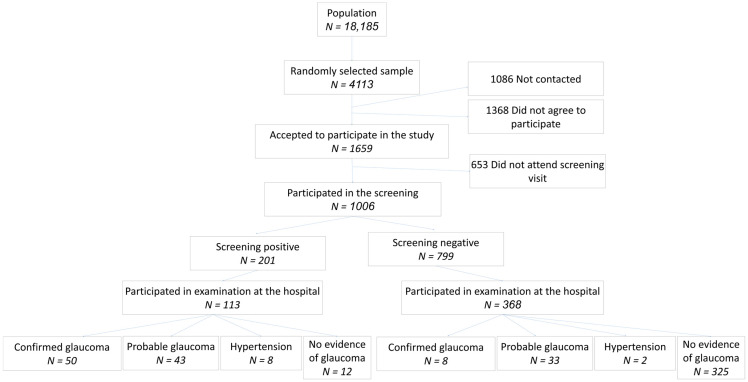
Distribution of the sample through the screening process. Flowchart with distribution of the sample through the screening process and the glaucoma consultation.

The random selection resulted in a list of 4113 individuals, but only 1006 (24.5%) were finally examined at the screening center (Figure 1). The demographic characteristics of the sample are summarized in Table 1 and described in more detail elsewhere [15]. The distribution between genders reflected the slightly larger number of females than males in the population, and approximately 40% were aged 55–64 years and 65–74 years, respectively, while 20% of the sample was older than 74 years. A total of 61 (5.1%) persons had been previously diagnosed with glaucoma, and 102 (10.1%) had a family history of glaucoma (Figure 2).

### 3.2. Image Quality

A non-significant tendency (*p* = 0.09) toward a greater percentage of fair/good quality images and useful images was obtained with OCT, 962 (97.2%) and 946 (94%), respectively, than with fundus photographs, 945 (95.5%) and 927 (92.1%), respectively. A total of 38 (34.5%) poor quality images corresponded to participants aged more than 74 years, while 31 (28.2%) were from younger participants. The frequency of poor-quality images increased significantly with increasing participant age (*p* < 0.0001).

### 3.3. Screening Results

The screening program identified 201 (19.9%) patients with suspicion of glaucoma, and 6 could not be assessed due to insufficient image quality. According to the univariate analysis, the significant baseline risk factors for glaucoma suspicion were older age, higher intraocular pressure (IOP), low visual acuity, and a personal record of ocular hypertension and retinal surgery (Table 1). Of all the patients identified as glaucoma suspects, 41 (20.4%) were identified in photographs only, 24 (11.9%) were identified in OCT images only, and 93 cases (46.3%) were identified in both types of images. In 43 (21.4%) participants, both tests were classified individually as normal by the evaluators, but DYSEO tagged them as “glaucoma suspects”. This occurred if any single glaucomatous sign was marked as present by the evaluator, and/or there was a C/D asymmetry of more than 0.3 between the two eyes, and/or the IOP was higher than 21 mmHg.

### 3.4. Detection of Other Diseases

Evaluators also identified 31 patients (3.1%) with signs of diabetic retinopathy (DR) and 155 patients (15.4%) with signs compatible with age-related macular degeneration (ARMD). Overall, 2 patients were suspected to have all three diseases, 11 were suspected to have glaucoma and DR simultaneously, 34 had signs of glaucoma and ARMD, and 7 were glaucoma suspects with signs of DR.

### 3.5. Examination at Glaucoma Clinic

All participants attending the screening phase of the study (*n* = 1006) were invited to participate in the study. A total of 481 (47.8%) accepted and were examined at the glaucoma clinic. Among these, reliable and reproducible visual fields could only be performed in 345 (71.7%). Confirmed glaucoma was detected in 58 (12.1%), possible glaucoma in 76 (15.8%), OHT in 10 (2.1%), findings other than glaucoma in 105 (21.8%), and no abnormalities in 232 (48.2%). (Figure 2) The stage of glaucoma in patients who were classified as negative at screening, but identified glaucoma or probable glaucoma at the glaucoma clinic, was initial (MD > −6 dB) in 80.5% (33 patients) and moderate (MD −11.9 to −6 dB) in 18.2% (6 patients). No advanced cases (MD < −11.9 dB) were identified among those negative at screening. The degree of glaucoma in all cases of confirmed glaucoma and probable glaucoma, among those who were positive at screening, is detailed in Table 2.

The anterior segment exam identified 3 cases with pseudoexfoliation (two syndromes and one glaucoma) and 17 cases with occludable angle (*n* = 7 with grade 1 in Shaffer’s classification and *n* = 10 with grade 2) and no cases with a completely closed angle. Among those persons with occludable angles, two had confirmed glaucoma, seven were classified as probable glaucoma, and eight had no signs of glaucoma. 

Of the 481 persons who agreed to participate in the glaucoma consultation, 113 had been classified as positive at screening and 368 as negative. Of those with a positive screening result, 50 were finally classified as having glaucoma, 43 as having probable glaucoma, and 8 as having OHT. Accordingly, the detection rate was 4.9% if definition 1 was used and 9.2% if definition 2 was applied. Among patients classified as positive at screening, no evidence of glaucoma was found in only 12, tests were of insufficient quality in 6, and the results of the examination were normal in 6. In contrast, among persons with a negative screening result, the final classification was confirmed glaucoma in only eight, and none of them had advanced disease.

### 3.6. Classification Accuracy

The diagnostic accuracy of the screening classification is detailed in Table 3. Sensitivity ranged from 69.4% to 86.2% and specificity from 82.1% to 97.4%. As expected, a broader diagnostic definition increased sensitivity but decreased specificity, which was nevertheless good for all definitions (Table 3). The negative predictive value was high for all definitions, while the positive predictive value was low for the detection of confirmed glaucoma with both structural and functional damage, but was 86% for the detection of glaucoma with functional or structural damage, and was above 94% for glaucoma patients and glaucoma suspects globally. 

### 3.7. Predisposing Factors for Glaucoma

The diagnosis of glaucoma was significantly associated with greater age for both definitions 1 (*p* = 0.02) and 2 (*p* = 0.03) but was not statistically significant for definition 3. Neither gender nor family history were associated with glaucoma diagnosis. Gonioscopy was not associated with diagnosis when we used definition 1 or 2, but an angle of 1 (Shaeffer’s classification) was associated with a greater chance of high IOP (*p* = 0.0002). 

## 4. Discussion

A discussion on the optimal tests for glaucoma screening is ongoing. A 2008 systematic review and meta-analysis on different screening tests for glaucoma found no single test or combination of tests that was significantly superior to the others [6]. A Delphi analysis on glaucoma screening performed in 2012 suggested that six different combinations of IOP measurements plus a functional test and a structural test seemed to be a good pairing for glaucoma screening, but none of them had optimal results [5]. More recently, a health technology assessment [9] confirmed the accuracy of imaging devices when used for identifying glaucoma cases in the community, together with tonometry and visual acuity, among persons referred to a specialized ophthalmic facility from community optometrists or general practitioners due to the possibility of glaucoma. In that study, Heidelberg retina tomograph (HRT) and glaucoma diagnostics (GDx) still seemed slightly more precise than OCT, but a meta-analysis performed by our group some years later, which included more recent versions of OCT, found that the sensitivity of OCT was similar to that of the other two imaging instruments but that specificity was significantly higher [8]. The superiority of OCT over other imagine devices was also found in a systematic review [7] and in a population-based study [16]. This was not surprising since the development of HRT and GDx was stopped some years ago, whereas OCT has been continuously improving in the last few years from time domain to spectral domain and swept source. 

During the screening process using OCT iVue, images of the NFL and the GCC were obtained and considered by the evaluators; nevertheless, ganglion cell layer data were not obtained during the glaucoma evaluation at the hospital for several reasons. First, the available Cirrus OCT did not measure GGC but only ganglion cell layer thickness. Second, two thorough studies, one systematic review [7] and one metanalysis [8], have demonstrated that ganglion cell layer measurements do not improve the sensitivity of NFL measurements for glaucoma detection. Although GCC images were taken during the screening program because of the ease of use of OCT iVue and the novelty of the usage of those parameters at the time when the study was designed some years ago, the evidence available does not recommend adding these measurements to NFL thickness for detection purposes.

Among people older than 55 years, the combination of OCT images and fundus photographs yielded a detection rate of 9.2% among those with either confirmed glaucoma or probable glaucoma (definition 2) and a rate of 4.9% if only confirmed glaucoma (definition 1) was used. These values are somewhat higher than those obtained in similar conditions by our own group with with HRT and GDx (4.1%) among people older than 40 years [10] Ohkubo et al. [17] with HRT (3.9%) or Mul et al. [18] with GDx (4.6%). 

More recently, a study evaluating the diagnostic accuracy of OCT for glaucoma detection in a population-based sample obtained high areas under the receiving operating characteristic curve of 0.86–0.94, and the detection rates, calculated from data, ranged from 3.2% to 3.7%, depending on the parameter used [16]. The lower value than that in our study is probably due to the fact that that study included persons aged 40–80 years, while our sample consisted of persons older than 55 years of age who, as expected, had a higher prevalence of glaucoma.

A very relevant issue concerning the diagnostic accuracy of imaging devices and any other type of tests for screening purposes is the sample. Most published studies have been performed in a highly controlled environment, and their results are biased by the inclusion criteria. These studies have tended to overestimate the accuracy of the devices used because the groups compared were clearly, and artificially, differentiated by the inclusion criteria. Diagnostic precision studies performed in population-based samples more accurately reflect real-life situations and are much more effective in assessing the usefulness of any device for screening purposes. This is one of the strengths of the present study, because it was performed in a population-based and randomly selected sample. A few previous studies have evaluated an OCT-based glaucoma screening protocol in general populations at risk. One study obtained areas under the receiver characteristic curve (AROC) of over 0.800 for several OCT parameters and identified 16.3% of glaucomas, using a discriminant analysis algorithm based on OCT parameters, which is similar to the 19% glaucoma suspects found in our study [19].

Some factors have been found to strongly influence OCT measurements, including those in population-based studies, and need to be taken into account. A population-based study confirmed that age, sex, axial length, disc area, and signal strength are significant factors influencing OCT values and need to be considered when interpreting the results of this procedure [20]. Another study [21] confirmed that, as expected, the ethnic-based diversity of the structure of the optic disc and RNFL also influenced OCT results. The findings of these studies suggest that more specific normative databases would be ideal to optimize the screening abilities of OCT.

The diagnostic accuracy of this screening program with OCT and photos varied depending on the definition of glaucoma applied and may seem slightly low. However, these values were obtained under conditions very closely resembling the real-world environment. Specifically, this was a population-based study, with a random selection of the sample, conducted in a screening setting, and with a group of experts and also some less experienced evaluators. Most published studies have been performed with preselected participants in the highly specialized environment of glaucoma clinics and with selected experienced evaluators; these conditions may be optimal to evaluate instruments but are very different from those of a population-based screening campaign.

Additionally, the diagnostic accuracy of any study is completely dependent on the definition applied for the disease to be detected. Most published studies still use a glaucoma definition that requires functional and structural damage, but it is widely accepted that structural damage often precedes detectable functional damage and that some cases, although fewer, have initial functional damage with no detectable structural damage. With this in mind, it is reasonable to assess this and other screening strategies with a definition that includes not only persons with functional and structural damage but also those with only detectable structural or functional damage. Definition 2 applied in this study included the latter, together with persons with confirmed glaucoma, and obtained a reasonable sensitivity (0.69), very high specificity (0.94), and positive and negative predictive values of 0.85. 

Previous studies have shown that including OCT in telemedicine equipment may improve the results of classification [8] and the reproducibility of the assessment. A previous study found good global diagnostic accuracy but did not recommend the exclusive use of imaging devices (OCT, HRT, or GDx) because they could miss up to 5% of cases with advanced glaucoma [22]. Data from the Rotterdam Eye Study [23] suggested that a visual field test could be more sensitive than OCT in glaucoma detection since the best OCT parameter offered a sensitivity of only 53% in identifying glaucoma cases with abnormal visual fields. We believe that these calculations may be biased by several factors. First, half of the original sample could not be included due to a lack of visual field tests. Second, in screening campaigns, functional tests are very difficult to perform, as they are time consuming and require repetition and because reliable tests are obtained in only a low percentage of participants. In our study, reliable field tests were obtained in only 71% of participants who completed both parts of the study (screening and glaucoma visit) compared with 97% participants with good quality OCT images, and only a slightly lower percentage had good quality photos. These data support the fact that imaging devices are much easier to apply and suited for screening purposes.

Our results with photos and OCT showed a sensitivity of 100% for advanced glaucoma cases and a sensitivity of 69% with a specificity of 95% to identify confirmed or probable glaucomas, which could be the benefit of associating OCT with color retinographs. Additionally, during the screening process, 32% of participants had findings in either photographs or OCT but not in both, suggesting the combination of both is more effective for screening purposes. Overall, the results confirmed good diagnostic accuracy in a real-world screening environment and support the use of imaging devices for glaucoma screening.

The present study was not designed as an epidemiological study, and the methods were aimed to meticulously identify glaucomatous optic neuropathy, but not so specifically defined to objectively identify findings (i.e., photographs) in the angle or the anterior segment. Nevertheless, anterior segment findings, including pseudoexfoliation and angle characteristics, were recorded by ophthalmologists in the electronic clinical chart, and 2 persons with pseudoexfoliation glaucoma and 17 with occludable angles were detected. Although the screening exam did not include, for obvious reasons, iridocorneal angle exanimation, this screening program based on IOP and imaging devices did help to identify persons with suspicion for angle closure.

Additionally, at the glaucoma examination, 13 persons had intraocular pressure over 21 mmHg in at least on eye. Of those, four had confirmed glaucoma, one was classified as probable glaucoma, and eight were ocular hypertensives. The apparently low percentage of cases with high pressure may be due to several reasons. Firstly, persons with high pressure, with or without glaucoma, are most likely to be diagnosed by an opportunistic finding since tonometers are generally used by ophthalmologists, but also by optometrists and optic shops, and, consequently, those persons are less likely to participate in these type of detection programs. Secondly, some persons identified as suspects during screening could have initiated treatment before attending the glaucoma exam at the hospital, which took place months after the screening process. Finally, since diurnal curves were not performed, it is not possible to conclude with precision how many cases of low- and high-pressure glaucoma were identified. Nevertheless, normal tension glaucoma was undoubtedly frequent, probably the majority, among those persons detected to have glaucoma in this screening program. 

This study has some limitations, which, we believe, did not preclude it from achieving its objectives and providing useful new data for glaucoma screening. First, the percentage of patients who could not be reached by telephone, as well as patient dropout, was high. Although this was not surprising for a population-based study, it could have affected the characteristics of the randomly selected sample. However, when comparing age and gender distribution of the final sample obtained to that of the original population, we found no statistically significant differences. Second, the willingness to design and assess a detection program in screening conditions (i.e., aimed at the general population, with random selection, and variation in operator experience) probably led to less accurate results than would have been obtained in a more controlled study, but it certainly more closely resembled a real clinical screening environment. Finally, cost and effectiveness need to be estimated in any screening strategy and are part of an ongoing study. 

In summary, the screening program based on OCT and fundus photographs identified almost 20% of glaucoma suspects among persons initially examined. The detection rate for confirmed glaucoma was 4.9% and was 9.2% for confirmed or probable glaucoma. Diagnostic accuracy was moderate to high, depending on the definition applied, but it was probably a good estimate for future population-based screening campaigns. 

## Figures and Tables

**Table 1:** Sample characteristics.

Categorical Variable	Categories	Total Group (*n* = 1006)	Suspects (*n* = 201) (19.9%)	Non-Suspects (*n* = 799) (79.4%)	*p*-Value
Gender	Female	523 (51.9)	94 (46.8)	426 (52.9)	0.2503
	Male	483 (48.1)	107 (53.2)	373 (47.1)	
Age	<65	425 (42.2)	64 (31.8)	360 (45.1)	<0.001
	65–74	386 (38.4)	81 (40.3)	305 (38.2)	
	>74	195 (19.4)	56 (27.9)	134 (16.7)	
Visual acuity	Low (<0.2)	63 (6.3)	31 (15.4)	33 (4.1)	<0.001
	Medium ([0.2–0.5])	166 (16.5)	41 (20.4)	119 (14.8)	
	High (>0.5)	777 (77.2)	129 (64.2)	647 (81.1)	
Intraocular pressure	[0–21]	979 (97.3)	178 (88.6)	795 (99.5)	<0.001
	>21	27 (2.7)	23 (11.4)	4 (0.5)	
Family history of glaucoma	yes	102 (10.1)	13 (6.5)	89 (11.1)	0.0504
Personal record of glaucoma	yes	61 (5.1)	24 (11.9)	34 (4.3)	<0.001

The screening program identified 201 (19.9%) persons with suspicion of glaucoma during the screening process. Six persons could not be assessed due to absence of useful images. Glaucoma suspects were older; had lower visual acuity; and, as expected, more frequently had a personal history of ocular hypertension or glaucoma.

**Table 2:** Degree of glaucoma according to mean visual field defect.

Degree of Visual Field Damage	Cases of Confirmed or Probable Glaucoma
Advanced (MD < −11.9 dB)	10 (10.7%)
Moderate (MD −11.9 to −6 dB)	15 (16.1%)
Initial (MD > −6 dB)	58 (62.3%)
No reliable visual field available	10 (10.7%)
Total	93

Degree of glaucoma based on mean defect (MD) of the visual field. For this calculation, only cases of confirmed glaucoma and probable glaucoma were included. Most of glaucomas identified by the screening program had initial glaucomatous damage. VF: visual field, dB: decibels.

**Table 3:** Diagnostic accuracy of screening program for detecting glaucoma.

	Sensitivity	Specificity	Negative Predictive Value	Positive Predictive Value
Definition 1				
Sample	0.862	0.821	0.970	0.467
<64 Years	0.684	0.874	0.949	0.448
65–74 Years	0.895	0.773	0.981	0.362
>74 Years	1.000	0.814	0.975	0.614
Initial glaucoma	0.813	0.834	0.972	0.382
Moderate glaucoma	0.857	0.800	0.888	0.750
Advanced glaucoma	1.000	0.888	0.874	0.815
**Definition 2**				
Sample	0.694	0.942	0.848	0.869
<64 years	0.587	0.980	0.838	0.931
65–74 years	0.720	0.891	0.865	0.765
>74 years	0.789	0.976	0.833	0.968
Initial glaucoma	0.673	0.948	0.848	0.853
Moderate glaucoma	0.714	0.923	0.667	0.938
Advanced glaucoma	1.000	1.000	0.868	0.898
**Definition 3**				
Sample	0.701	0.974	0.840	0.944
<64 years	0.571	0.990	0.821	0.966
65–74 years	0.754	0.957	0.865	0.915
>74 years	0.789	0.976	0.833	0.968
Initial glaucoma	0.643	0.973	0.839	0.926
Moderate glaucoma	0.727	1.000	0.625	0.933
Advanced glaucoma	1.000	1.000	0.868	0.898

Three global definitions of diagnosis were applied: confirmed glaucoma (definition 1); confirmed glaucoma or glaucoma suspect (definition 2), and confirmed glaucoma or glaucoma suspect or OHT (definition 3). As expected, sensitivity and specificity were higher with greater glaucomatous damage.

## Data Availability

Data are safely kept and anonymized and have not been made available.

## References

[1] Tham Y.C., Li X., Wong T.Y., Quigley H.A., Aung T., Cheng C.Y. (2014). Global Prevalence of Glaucoma and Projections of Glaucoma Burden through 2040: A Systematic Review and Meta-Analysis. Ophthalmology.

[2] Antón A., Andrada M.T., Mujica V., Calle M.A., Portela J., Mayo A. (2004). Prevalence of Primary Open-Angle Glaucoma in a Spanish Population: The Segovia Study. J. Glaucoma.

[3] Mitchell P., Smith W., Attebo K., Healey P.R. (1996). Prevalence of Open-Angle Glaucoma in Australia: The Blue Mountains Eye Study. Ophthalmology.

[4] Klein B.E.K., Klein R., Sponsel W.E., Franke T., Cantor L.B., Martone J., Menage M.J. (1992). Prevalence of Glaucoma: The Beaver Dam Eye Study. Ophthalmology.

[5] Burr J., Mowatt G., Hernández R., Siddiqui M., Cook J., Lourenco T., Ramsay C., Vale L., Fraser C., Azuara-Blanco A. (2007). The Clinical Effectiveness and Cost-Effectiveness of Screening for Open Angle Glaucoma: A Systematic Review and Economic Evaluation. Health Technol. Assess..

[6] Mowatt G., Burr J.M., Cook J.A., Siddiqui M.A.R., Ramsay C., Fraser C., Azuara-Blanco A., Deeks J.J. (2008). Screening Tests for Detecting Open-Angle Glaucoma: Systematic Review and Meta-Analysis. Investig. Opthalmol. Vis. Sci..

[7] Michelessi M., Lucenteforte E., Oddone F., Brazzelli M., Parravano M., Franchi S., Ng S.M., Virgili G. (2015). Optic Nerve Head and Fibre Layer Imaging for Diagnosing Glaucoma. Cochrane Database Syst. Rev..

[8] Fallon M., Valero O., Pazos M., Antón A. (2017). Diagnostic Accuracy of Imaging Devices in Glaucoma: A Meta-Analysis. Surv. Ophthalmol..

[9] Azuara-Blanco A., Banister K., Boachie C., Mcmeekin P., Gray J., Burr J., Bourne R., Garway-Heath D., Batterbury M., Hernández R. (2016). Automated Imaging Technologies for the Diagnosis of Glaucoma: A Comparative Diagnostic Study for the Evaluation of the Diagnostic Accuracy, Performance as Triage Tests and Cost-Effectiveness (GATE Study). Health Technol. Assess..

[10] Anton A., Fallon M., Cots F., Sebastian M.A., Morilla-Grasa A., Mojal S., Castells X. (2017). Cost and Detection Rate of Glaucoma Screening with Imaging Devices in a Primary Care Center. Clin. Ophthalmol..

[11] Thomas S.M., Jeyaraman M., Hodge W.G., Hutnik C., Costella J., Malvankar-Mehta M.S. (2014). The Effectiveness of Teleglaucoma versus In-Patient Examination for Glaucoma Screening: A Systematic Review and Meta-Analysis. PLoS ONE.

[12] Beardsley R., Law S.K., Caprioli J., Coleman A.L., Nouri-Mahdavi K., Hubschman J.-P., Schwartz S.D., Giaconi J.A., Parker A. (2017). Comparison of Outcomes between Endoscopic and Transcleral Cyclophotocoagulation. Vision.

[13] Kurysheva N.I., Pechenkina A.A., Goncharova A.S. (2021). Examination of Patients with Glaucoma during the COVID-19 Pandemic. Vestn. Oftalmol..

[14] Heidinger A., Falb T., Werkl P., List W., Hoeflechner L., Riedl R., Ivastinovic D., Hommer A., Lindner E. (2021). The Impact of Tape Sealing Face Masks on Visual Field Scores in the Era of COVID-19—A Randomized Cross-over Study. J. Glaucoma.

[15] Anton A., Nolivos K., Pazos M., Fatti G., Herranz A., Ayala-Fuentes M., Martínez-Prats E., Peral O., Vega-Lopez Z., Monleon-Getino A. (2021). Interobserver and Intertest Agreement in Telemedicine Glaucoma Screening with Optic Disk Photos and Optical Coherence Tomography. J. Clin. Med..

[16] Koh V., Tham Y.-C., Cheung C.Y., Mani B., Wong T.Y., Aung T., Cheng C.-Y. (2018). Diagnostic Accuracy of Macular Ganglion Cell-Inner Plexiform Layer Thickness for Glaucoma Detection in a Population-Based Study: Comparison with Optic Nerve Head Imaging Parameters. PLoS ONE.

[17] Ohkubo S., Takeda H., Higashide T., Sasaki T., Sugiyama K. (2007). A Pilot Study to Detect Glaucoma with Confocal Scanning Laser Ophthalmoscopy Compared with Nonmydriatic Stereoscopic Photography in a Community Health Screening. J. Glaucoma.

[18] Gaasterland D.E., Ederer F., Beck A., Costarides A., Leef D., Closek J., Banks J., Jackson S., Moore K., Vela A. (2000). The Advanced Glaucoma Intervention Study (AGIS): 7. The Relationship between Control of Intraocular Pressure and Visual Field Deterioration. Am. J. Ophthalmol..

[19] Nakano T., Hayashi T., Nakagawa T., Honda T., Owada S., Endo H., Tatemichi M. (2017). Applicability of Automatic Spectral Domain Optical Coherence Tomography for Glaucoma Mass Screening. Clin. Ophthalmol..

[20] Ramyashri S., Rao H.L., Jonnadula G.B., Addepalli U.K., Choudhari N., Senthil S., Garudadri C. (2021). Determinants of Optical Coherence Tomography Parameters in a Population-Based Study. Am. J. Ophthalmol..

[21] Tham Y.C., Chee M.L., Dai W., Lim Z.W., Majithia S., Siantar R., Thakur S., Rim T., Cheung C.Y., Sabanayagam C. (2020). Profiles of Ganglion Cell-Inner Plexiform Layer Thickness in a Multi-Ethnic Asian Population: The Singapore Epidemiology of Eye Diseases Study. Ophthalmology.

[22] Banister K., Boachie C., Bourne R., Cook J., Burr J.M., Ramsay C., Garway-Heath D., Gray J., McMeekin P., Hernández R. (2016). Can Automated Imaging for Optic Disc and Retinal Nerve Fiber Layer Analysis Aid Glaucoma Detection?. Ophthalmology.

[23] Springelkamp H., Lee K., Wolfs R.C.W., Buitendijk G.H.S., Ramdas W.D., Hofman A., Vingerling J.R., Klaver C.C.W., Abràmoff M.D., Jansonius N.M. (2014). Population-Based Evaluation of Retinal Nerve Fiber Layer, Retinal Ganglion Cell Layer, and Inner Plexiform Layer as a Diagnostic Tool for Glaucoma. Investig. Ophthalmol. Vis. Sci..

